# Associations and Impact Factors between Living Arrangements and Functional Disability among Older Chinese Adults

**DOI:** 10.1371/journal.pone.0053879

**Published:** 2013-01-14

**Authors:** Hui Wang, Kun Chen, Yifeng Pan, Fangyuan Jing, He Liu

**Affiliations:** 1 Department of Epidemiology and Health Statistics, Zhejiang University School of Public Health, Hangzhou, China; 2 Zhejiang Provincial Centre for Disease Control and Prevention, Hangzhou, China; Marienhospital Herne - University of Bochum, Germany

## Abstract

**Objectives:**

To examine the association of living arrangements with functional disability among older persons and explore the mediation of impact factors on the relationship.

**Design:**

Cross-sectional analysis using data from Healthy Aging study in Zhejiang Province.

**Participants:**

Analyzed sample was drawn from a representative rural population of older persons in Wuyi County, Zhejiang Province, including 1542 participants aged 60 and over in the second wave of the study.

**Measurements:**

Living arrangements, background, functional disability, self-rated health, number of diseases, along with contemporaneous circumstances including income, social support (physical assistance and emotional support). Instrument was Activities of Daily Living (ADL) scale, including Basic Activities Daily Living (BADL) and Instrumental Activities of Daily Living (IADL).

**Results:**

Living arrangements were significantly associated with BADL, IADL and ADL disability. Married persons living with or without children were more advantaged on all three dimensions of functional disability. Unmarried older adults living with children only had the worst functional status, even after controlling for background, social support, income and health status variables (compared with the unmarried living alone, ß for BADL: −1.262, ß for IADL: −2.112, ß for ADL: −3.388; compared with the married living with children only, ß for BADL: −1.166, ß for IADL: −2.723, ß for ADL: −3.902). In addition, older adults without difficulty in receiving emotional support, in excellent health and with advanced age had significantly better BADL, IADL and ADL function. However, a statistically significant association between physical assistance and functional disability was not found.

**Conclusion:**

Functional disabilities vary by living arrangements with different patterns and other factors. Our results highlight the association of unmarried elders living with children only and functioning decline comparing with other types. Our study implies policy makers should pay closer attention to unmarried elders living with children in community. Community service especially emotional support such as psychological counseling is important social support and should be improved.

## Introduction

Living arrangements are defined by household composition or the number and identity of the cohabitants. Household is a major factor in determining the social roles of the elderly by providing social integration, social support and interactions [1]. The social ties based on a person’s household situation bring instrumental, informational, and emotional supports from household members, just as with any other social tie [2], [3]. The social support theory, addressing both the structure and the interactions in relationships, proposes that strengthening social support can improve health and mediate the negative effects of stress [4]. Many previous studies have shown that social support positively influences health and reduces mortality [5]–[11].

However, social relations in household living arrangements are different from other social relations. There are expectations and obligations associated with family roles that shift over the course of life. Household members provide each other personal care, comfort and intimacy as well as aggravation and conflict [2]. Studies have reported that older adults with a concordance between their needs and environment have higher morale [12]. Living arrangement concordance based on the family household is closely connected with cohabitation status, living with/without a partner and marital status. Cohabitation status and marital status are important aspects of an individual’s social relations but not identical entities. Many studies separately use cohabitation status and marital status as predictors in the analyses of the association between living arrangements and health outcomes, but at present, findings are conflicting [13]–[20]. Thus, we argue that households with different structures for the number and identity of members make very different demands on the older adults in them and offer very different resources. If so, we should also see patterned differences in health among older persons living in various types of households. Following our theoretical emphasis on the qualitative differences in resources and demands among households of different structures, we compared health among persons living in different types of households in various combinations of cohabitation and marital status.

Living arrangements are closely related to the health and well-being of the older adults [21]. The associations between living arrangements and health outcomes, such as mortality, activities of daily living (ADL) disability, self-rated health, and psychological well-being have been reported by scholars [22]–[32]. However, the literature is not clear on the associations between living arrangements and the health or well-being of older adults. In China, there is a deep-seated tradition of coresidence with one or more married children stemming from the Confucian ideals of filial piety, but this tradition has declined over time as family sizes have decreased due to the one-child policy along with other social and economic changes [28]. China is facing enormous challenges of aging and the companying problem of services for the aged, especially older adults with disabilities. Disability is prevalent and costly among older adults. There are complex and multifactorial reasons for the development of such disabilities among older adults [33]–[35].

The purpose of this study was to investigate the association between functional disability and living arrangements in a rural sample of Chinese older adults in Zhejiang Province. In particular, we aimed to answer the following questions: (1) What are the relationships between living arrangements and basic activities daily living (BADL) disability, instrumental activities of daily living (IADL) disability, and activities of daily living (ADL) disability among older adults? (2) Do some impact factors such as socio-demographic characteristics, social support, self-rated health, and number of diseases mediate the associations between living arrangements and functional disability of older adults?

## Methods

### Participants and Procedure

Data for this paper were taken from the cross-sectional study on Healthy Aging in Zhejiang Province. It was a multidimensional survey of two random samples of the community-dwelling elderly in Hangzhou City (urban area) and Wuyi County (rural area), respectively. This study was approved by the Institutional Review Board of Zhejiang University. The reference population for this paper only included older adults registered at the family health center of the town of Wangzhai and Baimu in Wuyi County, in the southwestern region of Zhejiang Province. Data were collected from May to August 2011. Older adults who volunteered to participate in the study were selected according to the following criteria: (a) age≥60years and (b) living in Wangzhai or Baimu. All older adults, a total of 1724, registered at the health center were interviewed at home, informed regarding the objectives and procedures of the study, and invited to participate in the study. A total of 1565 older adults agreed to participate in the study by signing the informed consent form and receiving a gift. There were 23 participants later excluded for uncompleted or unreliable questionnaires, leaving a total of 1542 older adults, an effective response rate of 89.4%.

### Measures

#### Background

*Socio-demographic* variables included gender, age, education (illiterate, primary school or more).

#### Living arrangements

In view of largely mixed results of health benefits of living with others, particularly in regard to the relative benefits of coresidence with children versus living alone, the sample participants were classified into six types based on the number and identity of the cohabitants: unmarried persons living alone, married persons living alone, married persons living with spouse only, unmarried persons living with children, married persons living with children only, and married persons living with spouse and children. “Unmarried” in this paper referred to current status and included all types of non-married persons: widowers, the divorced and the never married.

#### Health

*Self-rated health* was designed to capture respondents’ subjective assessment of their own medical and functional status [2]. Respondents were asked to rate their health on a scale of poor, fair, good, and excellent; *Number of diseases* was assessed from reports noting what diseases from a list of 35 chronic diseases the participant had been diagnosed with (e.g., hypertension, diabetes, cancer).

#### Functional disability

Respondents were asked to self-report whether they had any difficulty with activities of daily living (*ADL*), including the basic activities of daily living (*BADL*) and instrumental activities of daily living (*IADL*) [36]. The *BADL* measure contained 6 items, including toileting, eating, dressing, grooming, general movement, and bathing on a four-point scale from “no difficulty” (1) to “complete disability” (4). The sum score for BADL ranged from 6 to 24.The IADL scale consisted of eight items: using the telephone, daily shopping, preparing meals, doing housekeeping, doing laundry, taking the bus, taking medicine, and handling personal finances. Each of these items was rated for the difficulty using a scale similar to that used for BADL (range: 8–32). The sum score for ADL reflecting global functional status [37] had a range from 14 to 56, combining of BADL and IADL. Lower scores indicated more intact functional abilities.

#### Contemporaneous circumstances

*Yearly personal income* was recorded as<5000, ≥5000 and <10000, or ≥10000 yuan per year. We focused on perceived social support comprising two subscales: physical assistance and emotional support. *Physical assistance* was measured using the question: “How much difficulty do you currently have if in acquiring physical assistance (e.g., money, goods, daily care)?” *Emotional support* was assessed by the question: “How much difficulty do you currently have if in need of emotional support (e.g., expressions of care, concern, affection and interest)?” Each question had a trichotomous answer: no difficulty, some difficulty, and severe difficulty.

### Statistical Analysis

Descriptive statistics were used for socio-demographic characterization, social support and health by calculating the proportion of distribution in each stratum of living arrangements. We used chi square (χ^2^) tests to identify group differences in proportions and one-way ANOVA F-tests for continuous variables. One-way ANOVA F-tests were applied to identify significant differences between BADL, IADL, and ADL and living arrangements, then an LSD post hoc multiple comparisons test was used to identify specific differences in the functional disability. To compare functional disability among the six types of living arrangements, we performed multiple linear regression analyses in two models, in which BADL disability, IADL disability and ADL disability served as the dependent variables, respectively. Model 1 was analyzed for each dependent variable, controlling for background variables (gender, age and education). Then, health variables (number of diseases and self-rated health) and contemporaneous circumstances (yearly personal income, physical assistance and emotional support) were added in Model 2 in addition to socio-demographic variables. A *P*≤0.05 was considered statistically significant. The data were analyzed using the Statistical Package for the Social Science (SPSS), version 17.0.

## Results

The descriptive characteristics of the study sample are shown in Table 1. Of the participants, the proportions of unmarried persons living alone, married persons living alone, older adults living with spouse only, unmarried persons living with children only, married persons living with children only, and the older adults living with spouse and children were 25.3%, 5.3%, 52.1%, 7.1%, 2.3%, and 7.8%, respectively. The proportion of females was more than double among unmarried persons living with children (75.5%) when compared to older adults living with their spouse and children (37.2%). Older adults living with a spouse and children (67.76±6.78 years) were significantly younger than unmarried persons living alone (76.32±7.62 years). Older participants living with their spouse and children had a much higher education level than married persons living with children only. The overwhelming majority of unmarried persons living alone (93.5%) had less than 5000 yuan of yearly personal income compared to 42% of those living with spouse and children. Many more married persons living with children only (77.1%) than married persons living alone (26.8%) had no difficulty in receiving physical assistance if in need. In addition, a greater number of older adults living with spouse and children (77.5%) than married persons living alone (31.7%) had no difficulty in getting emotional support if in need. The health status of unmarried persons living with children only was significantly worse. Fewer older adults living with spouse and children (37.8%) than unmarried persons living with children only (67.3%) perceived their health as fair or poor, and 82.9% of married persons living with children only had no chronic diseases compared to 50.0% of unmarried persons living with children only.

**Table 1:** Description of the study sample by living arrangements.

	Unmarried, alone (n = 390)	Married, alone (n = 82)	With spouse(n = 804)	Unmarried, with children (n = 110)	Married, withchildren (n = 35)	With spouse and children (n = 121)	*P*-value
Gender							<0.001
Male	128(17.8)	30(36.6)	438(54.5)	27(24.5)	20(57.1)	76(62.8)	
Female	262(67.2)	52(63.4)	366(45.5)	83(75.5)	15(42.9)	45(37.2)	
Age							<0.001
<80	218(55.9)	41(50.0)	626(77.9)	67(60.9)	24(68.6)	103(85.1)	
≥80	172(44.1)	41(50.0)	178(22.1)	43(39.1)	11(31.4)	18(14.9)	
Mean	76.32±7.62	74.95±8.04	70.32±7.33	75.81±8.13	68.15±7.89	67.76±6.78	<0.001
Education							<0.001
Illiterate	328(85.9)	63(77.8)	585(73.2)	103(93.6)	34(97.1)	72(61.5)	
Primary school or more	54(14.1)	18(22.2)	214(26.8)	7(6.4)	1(2.9)	45(38.5)	
Personal income (yuan per year)							<0.001
<5000	362(93.5)	68(84.0)	527(65.8)	83(75.5)	29(82.9)	50(42.0)	
≥5000 and <10000	21(5.4)	11(13.6)	183(22.8)	10(9.1)	2(5.7)	37(31.1)	
≥10000	4(1.0)	2(2.5)	91(11.4)	17(15.5)	4(11.4)	32(26.9)	
Physical assistance							<0.001
No difficulty	172(44.3)	22(26.8)	384(47.8)	59(54.1)	27(77.1)	89(74.2)	
Some difficulty	167(43.0)	55(67.1)	342(42.6)	38(34.9)	7(20.0)	23(19.2)	
Severe difficulty	49(12.6)	5(6.1)	77(9.6)	12(11.0)	1(2.9)	8(6.7)	
Emotional support							<0.001
No difficulty	165(42.7)	26(31.7)	412(51.8)	58(53.2)	27(77.1)	93(77.5)	
Some difficulty	159(41.2)	51(62.2)	321(40.3)	36(33.0)	6(17.1)	21(17.5)	
Severe difficulty	62(16.1)	5(6.1)	63(7.9)	15(13.8)	2(5.7)	6(5.0)	
Number of diseases							0.002
0	202(52.3)	52(63.4)	449(56.3)	55(50.0)	29(82.9)	81(68.6)	
1	134(34.7)	22(26.8)	267(33.5)	36(32.7)	4(11.4)	25(21.2)	
≥2	50(13.0)	8(9.8)	82(10.3)	19(17.3)	2(5.7)	12(10.2)	
Self-rated health							0.003
Excellent	47(12.1)	8(9.8)	90(11.3)	14(12.7)	2(5.7)	22(18.5)	
Good	136(35.0)	34(41.5)	283(35.4)	22(20.0)	18(51.4)	52(43.7)	
Fair	166(42.7)	34(41.5)	333(41.6)	57(51.8)	15(42.9)	34(28.6)	
Poor	40(10.3)	6(7.3)	94(11.8)	17(15.5)	0(0.0)	11(9.2)	

Table 2 presents the means and standard deviations of the continuous scores from the BADL, IADL, and ADL. Significant associations of functional disability with living arrangements are shown. Functional disability varied by living arrangements. Unmarried persons living with children only had the lowest scores on all three dimensions of functional disability. Furthermore, married persons living with children only had significantly better function than unmarried persons living alone, older adults living with spouse only and both with spouse and children in regards to IADL and ADL disability.

**Table 2:** Distribution and difference of functional disability by living arrangement.

		(1) Unmarried,alone	(2) Married,alone	(3) With spouse	(4) Unmarried, with children	(5) Married,with children	(6) With spouse and children	*P*-value*
BADL Score		6.80±1.98	6.28±1.42	6.75±2.44	7.76±3.94	6.00±0.00	6.61±1.65	**<0.001**
	(1)	–	0.070	0.743	**<0.001**	0.054	0.447	
	(2)	–	–	0.085	**<0.001**	0.554	0.324	
*P*-value#	(3)	–	–	–	**<0.001**	0.064	0.545	
	(4)	–	–	–	–	**<0.001**	**<0.001**	
	(5)	–	–	–	–	–	0.175	
IADL Score		10.71±4.22	9.65±3.16	10.26±4.28	12.29±6.51	8.17±0.86	10.24±4.71	**<0.001**
	(1)	–	0.046	0.099	**0.001**	**0.001**	0.302	
	(2)	–	–	0.226	**<0.001**	0.097	0.346	
*P*-value#	(3)	–	–	–	**<0.001**	**0.006**	0.953	
	(4)	–	–	–	–	**<0.001**	**0.001**	
	(5)	–	–	–	–	–	**0.014**	
ADL Score		17.51±5.95	15.93±4.26	17.01±6.49	20.05±10.20	14.17±0.86	16.97±6.35	**<0.001**
	(1)	–	0.046	0.2218	**<0.001**	**0.004**	0.423	
	(2)	–	–	0.150	**<0.001**	0.182	0.265	
*P*-value#	(3)	–	–	–	**<0.001**	**0.012**	0.940	
	(4)	–	–	–	–	**<0.001**	**<0.001**	
	(5)	–	–	–	–	–	**0.026**	

* *P*-value is for One-way ANOVA F-tests between BADL, IADL, and ADL and living arrangements.

# *P*-value is for an LSD post hoc multiple comparisons test.

Table 3 reports the results of the multivariate analysis of the associations between functional disability scores and living arrangements. The figures in the table are coefficients from multiple linear regressions of functional disability on sets of independent variables.

**Table 3:** Coefficients from multiple linear regression analyses of functional disability by living arrangement.

	BADL		IADL		ADL	
	Model 1	Model 2	Model 1	Model 2	Model 1	Model 2
Living arrangement						
Unmarried, children	Ref	Ref	Ref	Ref	Ref	Ref
Unmarried, alone	−0.916***	−0.917***	−1.451**	−1.401***	−2.370***	−2.326***
Married, alone	−1.397***	−1.262***	−2.429***	−2.112***	−3.833***	−3.388***
With spouse	−0.683**	−0.679**	−1.089*	−1.071**	−1.785**	−1.771**
With spouse, children	−0.616	−0.469	−0.543	−0.244	−1.061	−0.613
Married, with children	−1.646***	−1.166**	−3.730***	−2.723***	−5.380***	−3.902***
Gender						
female	Ref	Ref	Ref	Ref	Ref	Ref
Male	−0.058	−0.032	−0.296	−0.223	−0.344	−0.243
Age	0.071***	0.043***	0.206***	0.145***	0.275***	0.186***
Education						
primary school or more	Ref	Ref	Ref	Ref	Ref	Ref
Illiterate	0.385**	0.189	0.900***	0.460	1.256**	0.619
Personalincome (yuan per year)						
≥10000		Ref		Ref		Ref
≥5000 and <10000		−0.462*		−1.376***		−1.775**
<5000		−0.225		−0.669		−0.867
Physical assistance						
severe difficulty		Ref		Ref		Ref
Some difficulty		−0.068		0.613		0.680
No difficulty		−0.235		−0.034		−0.261
Emotional support						
severe difficulty		Ref		Ref		Ref
Some difficulty		−1.194***		−2.022***		−3.222***
No difficulty		−1.325***		−2.300***		−3.635***
Number of diseases						
≥2		Ref		Ref		Ref
1		−0.250		−0.421		−0.667
0		−0.297		−0.745*		−1.028*
Self-rated health						
poor		Ref		Ref		Ref
Fair		−2.059***		−4.533***		−6.597***
Good		−2.264***		−4.818***		−7.073***
Excellent		−2.431***		−5.529***		−7.981***

* *P*< = 0.05,

** *P*< = 0.01,

*** *P*< = 0.001.

The first panel presents the results for BADL disability. In Model 1, being older and a low level of education were independently associated with BADL disability. Unmarried persons living alone, married persons living alone or with children only, and older adults living with spouse only reported better BADL ability than unmarried persons living with children only. In Model 2, when controlling for contemporaneous circumstances, background and health variables, the magnitude of the associations did not change except that the coefficient for married persons living with children only decreased from −1.646 to −1.166. These results indicate that part of the association between living arrangements and BADL disability was due to the confounding effect of the added variables. The second panel presents the results for IADL disability. Here again, unmarried persons living with children only had significantly more IADL disability than older people in other living arrangements except for older adults living with spouse and children together. After controlling for other covariates, the coefficient decreased from −3.730 to −2.723, but the association still had statistical significance. In the third panel, the results for ADL disability were similar to those for BADL and IADL disability. In addition, the results in these three panels also indicate that older adults who had no difficulty in receiving emotional support and who were in excellent health have significantly better BADL, IADL and ADL function.

## Discussion

In this paper, our results implicates that the social context formed by the living arrangements based on family household has been shown to be important social etiology for health. Living arrangements are significantly related to functional disability among older adults. Compared with other types of living arrangements, unmarried persons including widowers, the divorced and the never married, living with children only are disadvantaged on all three dimensions of functional disability as measured by the BADL, IADL and ADL. Married persons living alone or with children only do appear to have better functionality. Our study suggests that persons living alone did not report worse functional status. In fact, an earlier study showed that urban older adult living with children and without spouses had worse outcomes as compared to those who lived alone [38]. Our results also are consistent with the findings of Hughes and Gove [39]. However, some previous studies found that living alone disadvantaged individuals in regards to functional health [40], [41] and other measures of health [40]. We explain this finding several ways. First, functional disability preceded cohabitation and older adults with functional disability may self-select to cohabitate with children in the absence of the spouse. Second, functional disability follows cohabitation status and unmarried adults may require more support than they receive from children. Thus, unmarried persons living with children experience demands that exceed their coping resources and this imbalance ultimately affects their health and even functional status.

Further, in our study living arrangements combining marriage and cohabitation status do not merely seem to reflect alternative status correlates with functional disability, respectively. Unmarried persons living with children only do appear to be the least healthy on all measures, which is partly consistent with the findings of Hughes et al. who found the association only in women aged 51–61 [2]. The findings in these two studies indicate that health can be impacted both by cohabitation and marital status. A couple of studies have reported on the independent effect of cohabitation status and marital status [2], [42].Our study implies that married status may show positive association with functionality. Many previous studies also reported having married persons experience better health. There are several explanations, such as a direct health promotional effect of the marriage, social support from spouse [43], [44], even the daily availability of health behaviors from another person [45].

However, we see substantial variation in the extent to which persons in the other living arrangements different from older adults in unmarried status and living with children. Older persons with similar marital status experience different functionality for different cohabitation status. So a protective effect of marriage that may be weakened by the identity and number of the partners. In this study, an unmarried status, including being widowed, divorced or never married reveals a passive single status. While, the cohabitation of married persons was typed into two statuses, one was living with spouse, the other was without spouse. In China, living with adult children is traditional form, which was generally replaced by living independently, or in “empty nests”. Married persons living without spouse in our study may tend to be a more active preference shaped not only by cultural norms but also by education and exposure to new ideas [46]. Some recent studies indicated that elders living with children mainly for daily care, or expectations that they “serve” younger generations [47]. The married elders living without spouse, but with children always play the latter role. In addition, the reasons for living without a spouse among married older adults also may be marital discord or independent status. The preference reflects the congruence model of the person-environment fit, with concordance of living arrangements predicting better health [21]. Anyhow, our results suggest living arrangements based on household structure are important beyond marital status–that in certain circumstances marriage does not protect health and being single does not damage health.

This study also provides evidence of association between socio-demographic, contemporaneous circumstances and health factors and functional disability. Emotional support as a type of social supports in our study appears to exhibit a significantly positive association with better functional status. It may contradict the findings that persons living alone had better functional status comparing with those who were unmarried elders living with children. In fact, 67.3% of the unmarried and living with children reported poor or fair self-rated health, while the proportion among the living alone with unmarried and married status just were 53.0% and 48.8%, respectively. The results of association between social support and functional status are in accordance with previous evidence from other studies [48]–[50]. However, few studies have stratified social support into physical assistance and emotional support. We think that the effects of physical assistance and emotional support on health are different because perceptions of emotional support tend to be correlated with marital status and the social composition of the household [2], [51], [52]. Interestingly, emotional support is positively associated with the functional status of older adults. However, such an association cannot be found between physical assistance and functional status. It is obvious that higher age is associated with functional status decline for an increase in disease. It is understandable that good ADL functioning shows positive correlates with good self-rated health.

There are limitations to this study. One limitation might be its cross-sectional design. We cannot draw conclusions of causality between living arrangements and functional status. It is possible, though unlikely, that the associations observed between unmarried elders living with children and functioning decline, the living alone and good functional status are a result of reverse causality. Second, another limitation may be that data on social support in this study relies on self-reported survey methods, not based on the professional scales. It was measured by the two dimensions of physical assistance and emotional support. Among previous studies, there is a lack of consistence concerning the content of social support, majority which is measured as a whole. In fact, four types of social support: instrumental, informational, emotional and appraisal support were defined by House [53]. Further study could focus on more exact classification and measurement. Third, living arrangements in this study only reflect the current situation, did not have information on the preference and satisfaction. Sarwariet al. emphasized that the advantage of living alone may reflect a preference. Because independent living expressed as a health benefit in terms of decreased functional reliance on others [18]. Lawton et al. pointed out that any observed association between living arrangements and health may have more to do with factors and characteristics that are idiosyncratic to the individual and her choice to live alone [17]. The absence of information regarding individual motivation limits our ability to identify the mechanism relating living arrangements to health. So longitudinal study with deep information -details of cohabitant children, individual reference and maintenance time and so on- may clarify the causality and mechanisms of association between living arrangements and health.

In conclusion, functional disabilities vary by living arrangements in different patterns and to different degrees. Our results highlight the association of unmarried elders living with children only and functioning decline comparing with other types. It implies policy makers should pay closer attention to unmarried elders living with children in community. Community service such as social support also should be improved including life care, sanitary, day nursing home, medical care, psychological counseling, rehabilitation and emergency assistance.
